# Inherent atomic mobility changes in carbocation intermediates during the sesterterpene cyclization cascade

**DOI:** 10.3762/bjoc.15.184

**Published:** 2019-08-07

**Authors:** Hajime Sato, Takaaki Mitsuhashi, Mami Yamazaki, Ikuro Abe, Masanobu Uchiyama

**Affiliations:** 1 Graduate School of Pharmaceutical Sciences, Chiba University, 1-8-1 Inohana, Chuo-ku, Chiba 260-8675, Japan; 2 Clustering of Pioneering Research (CPR) Advanced Elements Chemistry Laboratory, RIKEN, 2-1 Hirosawa, Wako, Saitama 351-0198, Japan; 3 Graduate School of Pharmaceutical Sciences, University of Tokyo, 7-3-1 Hongo, Bunkyo-ku, Tokyo 113-0033, Japan

**Keywords:** biosynthesis, carbocation, DFT, substrate recognition, terpene cyclase

## Abstract

We previously showed that the regio- and stereoselectivity in terpene-forming reactions are determined by the conformations of the carbocation intermediates, which reflect the initial conformation of the substrate, geranylfarnesyl diphosphate (GFPP). However, it remains unclear how the initial conformation of GFPP is controlled, and which part(s) of the GFPP molecule are important for its fixation inside the substrate-binding pocket. Here, we present the first detailed analysis of the inherent atomic mobility in carbocation intermediates during sesterterpene biosynthesis. We identified two methyl groups as the least mobile of all the carbons of the carbocation intermediates in the first half of the cyclization cascade. Our analysis suggests that these two methyl groups are critical for the preorganization of GFPP in the biosynthetic pathways leading to sesterfisherol and quiannulatene.

## Introduction

**Scheme 1:**
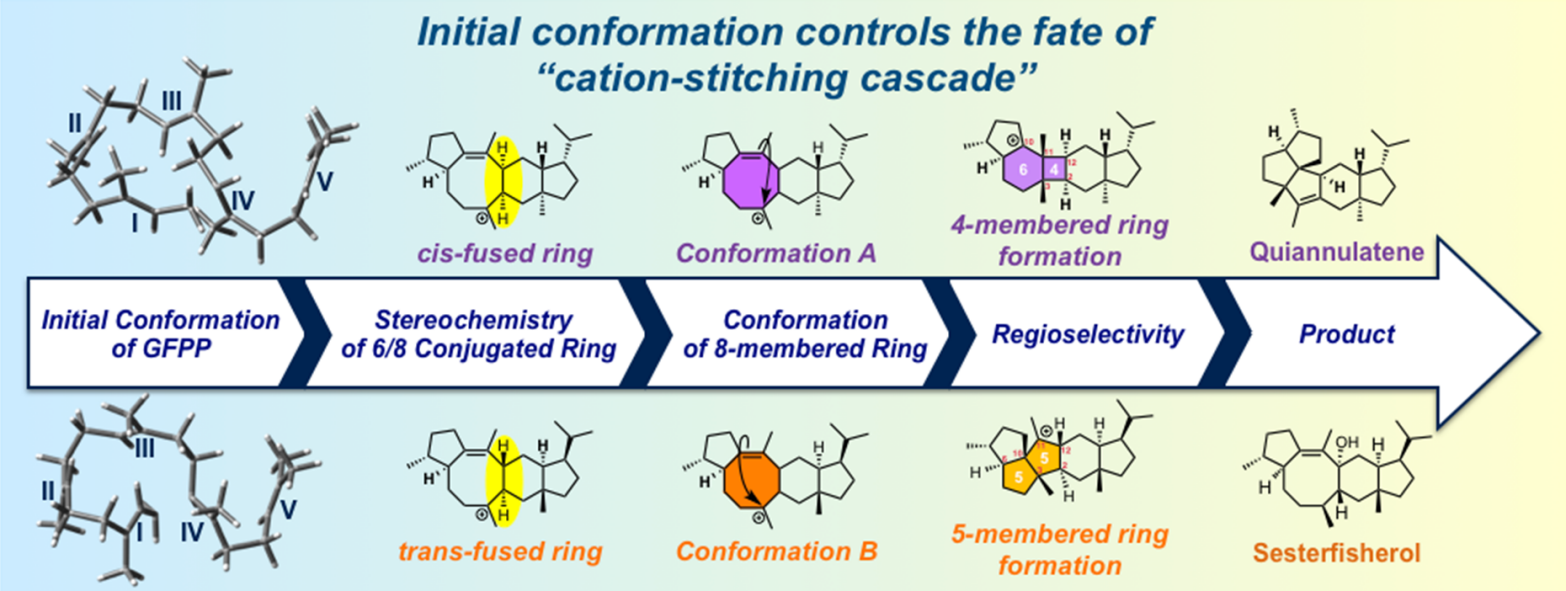
The regio- and stereoselectivity in quiannulatene and sesterfisherol biosynthesis are determined by the initial conformation of GFPP.

**Scheme 2:**
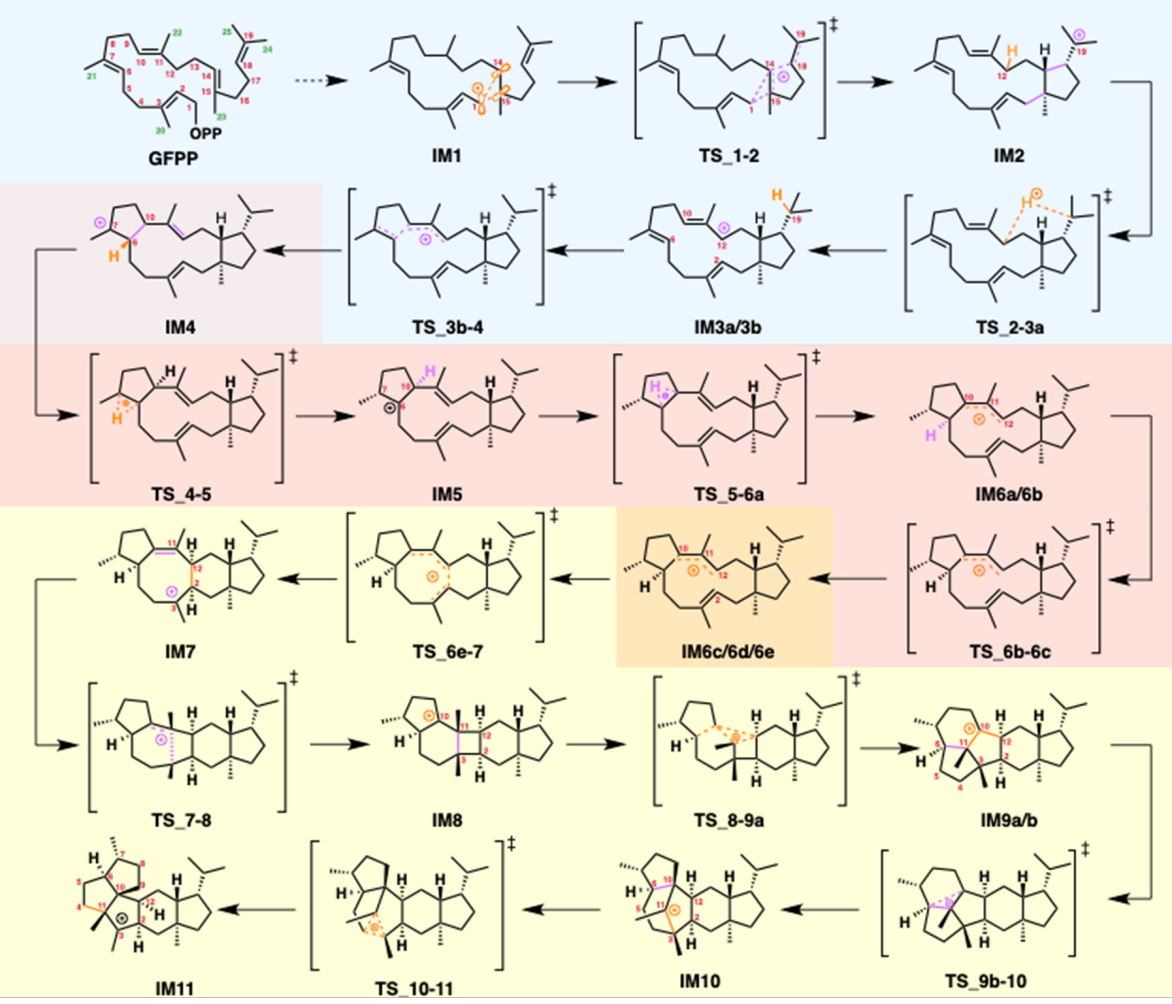
Reaction mechanism of quiannulatene biosynthesis. GFPP: geranylfarnesyl diphosphate, IM: intermediate. Quiannulatene is formed by the deprotonation of **IM11**. Phase (I): 5/12/5 tricycle formation is highlighted in blue. Phase (II): conformational changes and hydrogen shifts are highlighted in orange. Phase (III): ring rearrangements are highlighted in yellow.

**Scheme 3:**
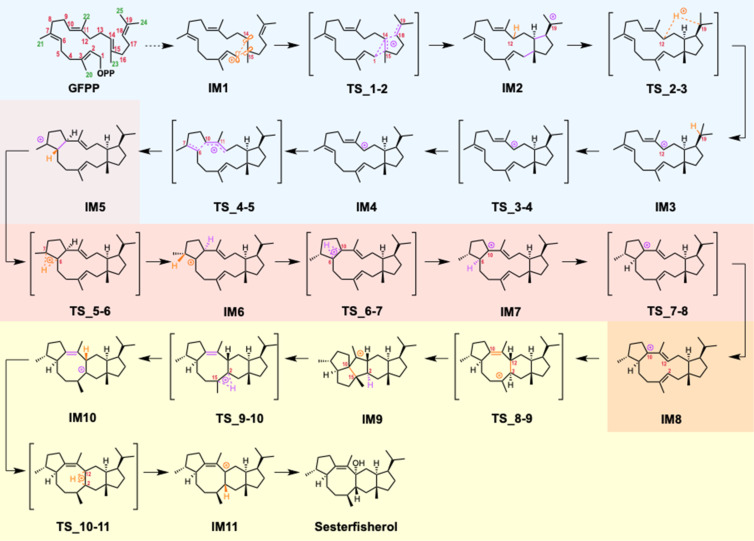
Reaction mechanisms of sesterfisherol biosynthesis. Sesterfisherol is formed by the hydration of **IM11**. Phase (I): 5/12/5 tricycle formation is highlighted in blue. Phase (II): conformational changes and hydrogen shifts are highlighted in orange. Phase (III): ring rearrangements are highlighted in yellow.

Terpene synthases are thought to have four main roles: (i) triggering the biosynthetic cyclization cascade by the elimination of pyrophosphate or by protonation; (ii) preorganization of the substrate to generate the reactive conformation; (iii) protection of reactive intermediates from water; and (iv) termination of the reaction by deprotonation or hydration. We previously established the importance of conformation in the carbocation cyclization cascade [1], focusing on two sesterterpenes, i.e., quiannulatene [1–2] and sesterfisherol [3–5], as representative examples. These two sesterterpenes are synthesized via a 5/12/5 tricyclic intermediate, which leads to a 5/6/8/5 tetracyclic intermediate. This, in turn, is transformed to a 4/6-membered ring in quiannulatene biosynthesis, whereas 5/5 ring formation proceeds in sesterfisherol biosynthesis (Scheme 1, Scheme 2, and Scheme 3). Based on our DFT calculations, this regioselectivity is determined by the conformation of the eight-membered ring in the 5/6/8/5 tetracyclic intermediate, which is derived from the initial conformation of GFPP. However, it remains unclear how the initial conformation of GFPP is controlled, and which part(s) of the GFPP molecule are most important for enzymatic preorganization in the terpene cyclase active site [3,5–6].

Although many terpene cyclases are known [6–10], it is still challenging to identify the precise initial conformation of the oligoprenyl diphosphate substrate in the active site, even by X-ray crystal structure determination. This is because the substrate can sometimes bind to the active site in an unreactive conformation [11]. Recently, Siegel and Tantillo reported an innovative method for predicting the docking mode of carbocation intermediates in terpene cyclase [12–13], based on QM calculation and computational docking with the Rosetta protein modeling suite [14–15]. Interestingly, probable docking modes are quite limited in the first few steps, but are much more diverse in the later part of the cyclization cascade, which may indicate that the affinity of carbocation intermediates for the terpene cyclase decreases as the reaction proceeds. Hence, we hypothesized that some part(s) of the substrate structure are less mobile (relatively fixed) in the first few steps of cyclization cascade, and thus play essential roles in the enzymatic preorganization of GFPP.

There have been many theoretical studies of terpene-forming reactions [16–18], and it appears that inherent reactivity [17] is in good accordance with the experimental outcome. This may mean that terpene cyclases do not tightly regulate the cyclization reaction steps once the carbocation is generated. Therefore, we considered that key regions of GFPP that control the fit of the substrate to the enzyme’s binding site could be identified by calculating the inherent structural mobility of the carbocation intermediates. This does involve the assumption that we can neglect the influence of changes in the interior structure of the enzyme as the reaction proceeds; however, based on the above-mentioned reports, we regard this as reasonable. Nevertheless, to minimize the effects of such changes, we focused on the first half of the cyclization cascade. In this study, we report the first analysis of the inherent structural mobility of carbocation intermediates in sesterterpene biosynthesis, and we discuss the implications for the mechanism of fixation (preorganization) of the substrate GFPP inside the binding pocket of the enzyme.

## Results and Discussion

**Figure 1:**
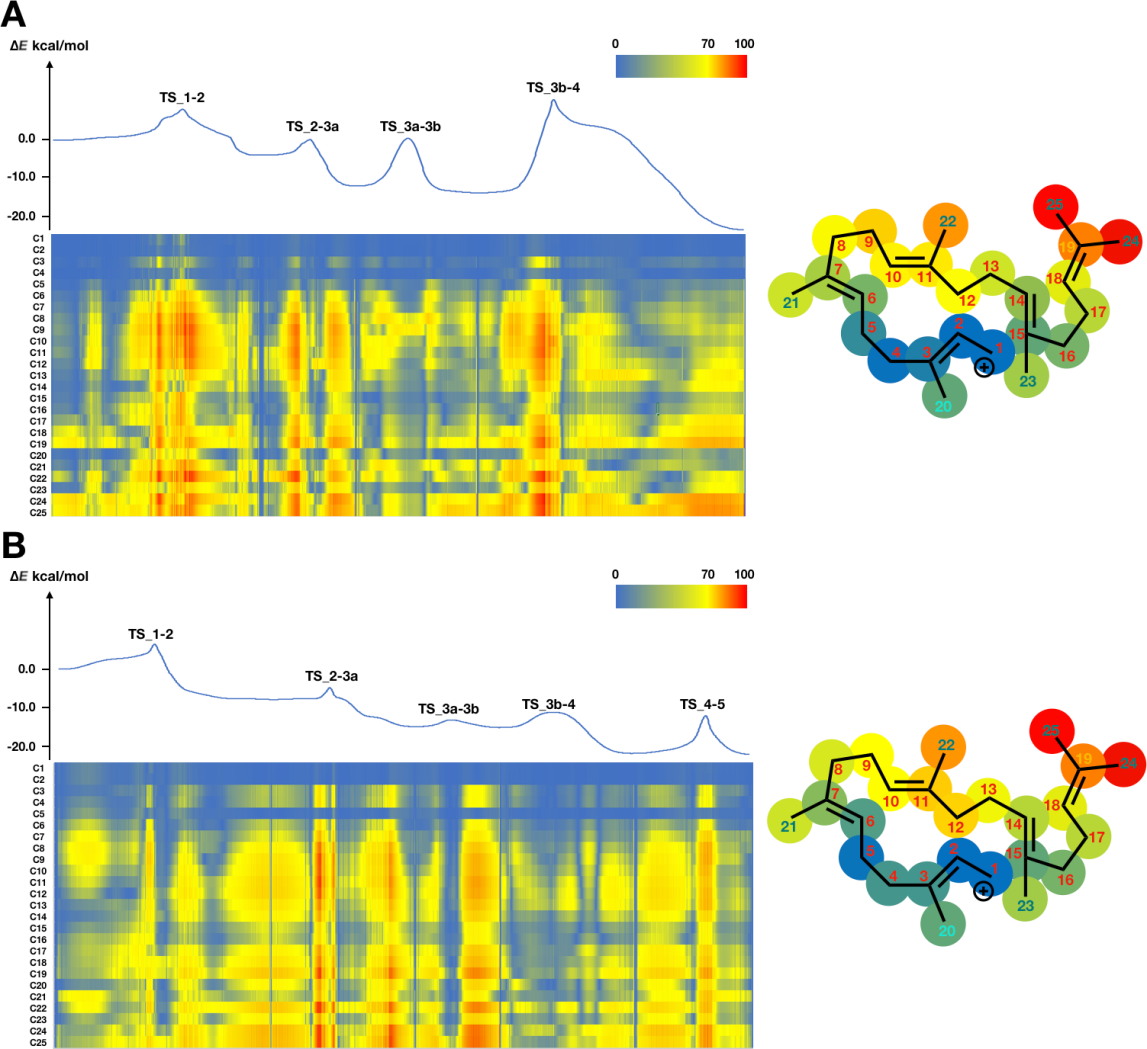
Energy diagram and heat map analysis of 5/12/5 tricycle formation (A) **IM1–IM4** in quiannulatene biosynthesis. (B) **IM1–IM5** in sesterfisherol biosynthesis. An energy diagram, heat map of inherent mobility and structural heat map are shown. All energies are shown in kcal/mol. The Y axis shows the carbon number and the X axis shows the coordinate in the heat map. The right structural heat map shows the total mobility in tricycle formation. Red means high mobility, yellow means moderate mobility, and blue means static. TS: transition state.

**Figure 2:**
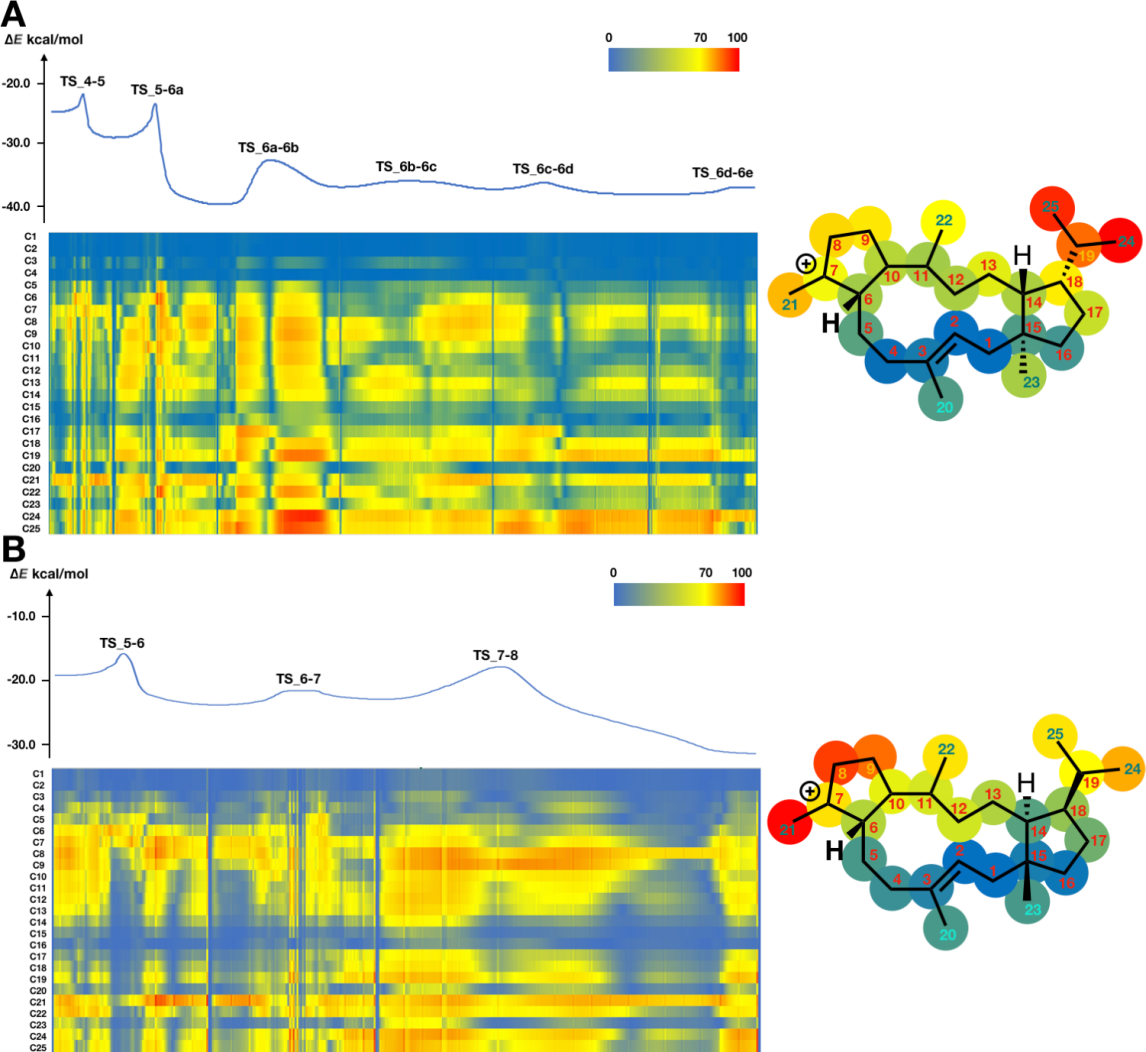
Energy diagram and heat map analysis of conformational change and hydrogen shift (A) **IM4–IM6e** in quiannulatene biosynthesis. (B) **IM5–IM8** in sesterfisherol biosynthesis. An energy diagram and heat map of inherent mobility are shown. All energies are shown in kcal/mol. The Y axis shows the carbon number and the X axis shows the coordinate in the heat map. The right structural heat map shows the total mobility. Red means high mobility, yellow means moderate mobility, and blue means static. TS: transition state.

**Figure 3:**
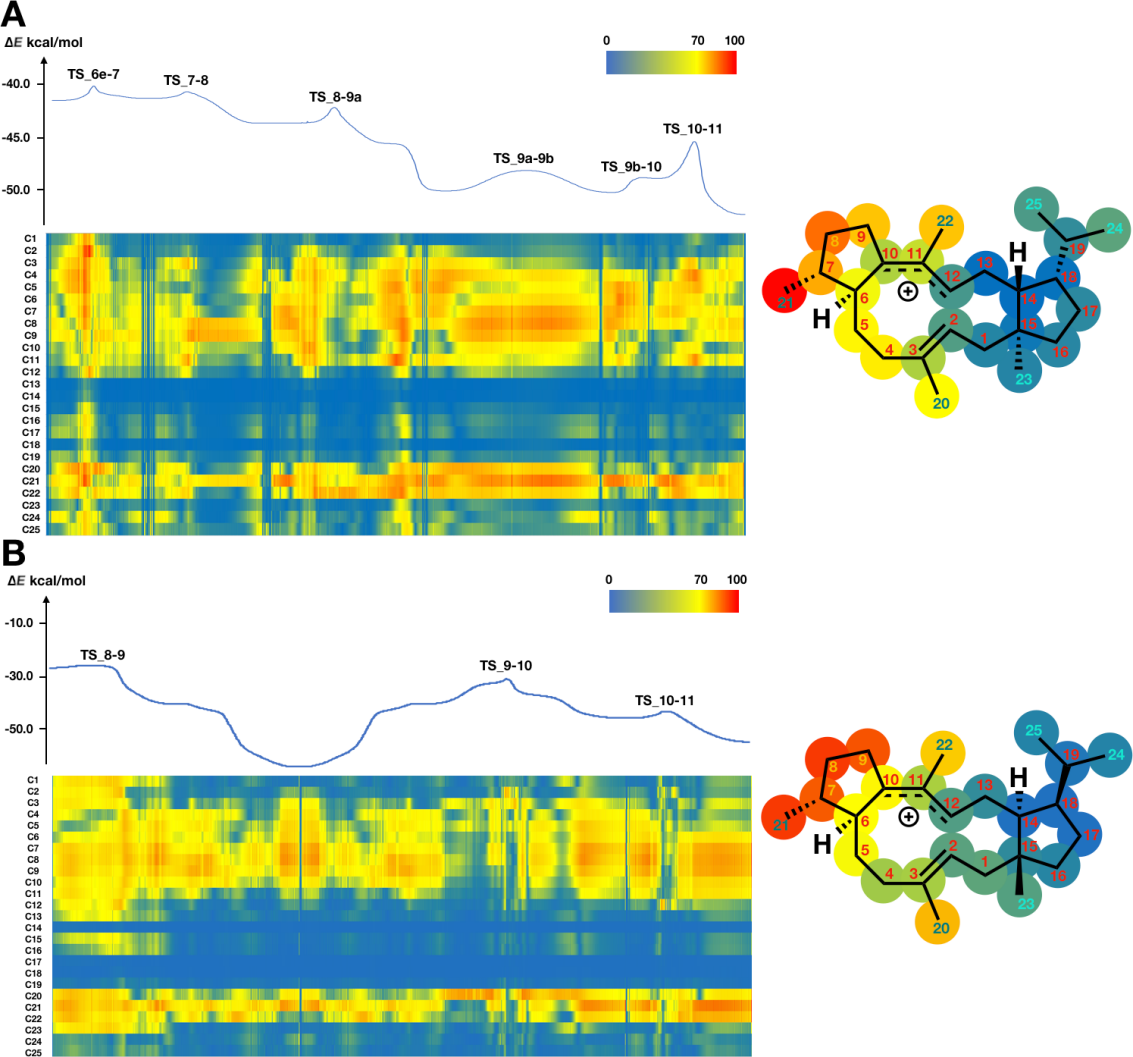
Energy diagram and heat map analysis of ring rearrangement (A) **IM6e–IM11** in quiannulatene biosynthesis. (B) **IM8–IM11** in sesterfisherol biosynthesis. An energy diagram and a heat map of inherent mobility are shown. All energies are shown in kcal/mol. The Y axis shows the carbon number and the X axis shows the coordinate in the heat map. The right structural heat map shows the total mobility. Red means high mobility, yellow means moderate mobility, and blue means static. TS: transition state.

For the analysis of inherent structural mobility, we firstly carried out IRC calculations using GRRM11 with Gaussian 09, obtaining 2609 plots for quiannulatene formation and 2640 plots for sesterfisherol formation. We divided the whole biosynthetic process into three phases; (I) 5/12/5 tricycle formation, (II) conformational changes and hydrogen shifts, and (III) ring rearrangements (Scheme 2 and Scheme 3). Then, we computed the inherent structural mobility of the carbocation intermediates by calculating the displacements of all carbons between each plot. The results of these analyses are shown as heat maps in Figure 1, Figure 2, and Figure 3. In these heat maps, the Y axis shows the carbon number and the X axis shows the coordinate. Highly mobile carbons are shown in red, moderately mobile carbons in yellow, and static carbons in blue.

### Phase I: 5/12/5 tricycle formation

Based on our inherent mobility analysis, C1–C5, C15, C16, C20, C21 and C23 are static during 5/12/5 tricyclic formation in the biosynthesis of both quiannulatene and sesterfisherol (Figure 1). Interestingly, although the conversion of **IM1** to **IM2** involves C1–C15 and C14–C18 bond formations, the displacements of C6–C12, and C22 are also large, indicating that these regions, though they are distant from the reaction centre, are relatively flexible and not tightly fixed by the enzyme. As we reported previously, the initial conformation of GFPP, in particular the orientation of six methyl groups (C20–C25), is critical. Therefore, we focused on these methyl groups. While the C20, C21 and C23 methyl groups are quite static in phase I, the other three methyl groups are relatively flexible, which suggests that C20, C21 and C23 could be key determinants of the affinity for the enzyme’s binding pocket. The C24 and C25 methyl groups are the most mobile moieties, and the dramatic displacements of these two methyl groups are consistent with a previous report that the counterclockwise rotation of the isopropyl moiety is necessary for 1,5-hydrogen shift to occur (**TS_2-3a** in quiannulatene biosynthesis and **TS_2-3** in sesterfisherol biosynthesis) [19].

### Phase II: conformational changes and hydrogen shifts

In phase II, different trends of inherent mobility are seen between quiannulatene and sesterfisherol biosynthesis. As shown in Figure 2, C1–C5, C15, C16, C20, and C23 are static, as in phase I. However, C21 is quite mobile, because the C7 carbocation becomes an sp^3^ carbon due to 1,2-hydrogen shift (**TS_4-5**). After two successive 1,2-hydrogen shifts, four-step conformational changes take place in quiannulatene biosynthesis, in which C24 and C25 are highly mobile. On the other hand, C8, C9 and C21 are quite mobile in sesterfisherol biosynthesis. These different mobility trends result from the different types of conformational change required to achieve regioselectivity in the following ring rearrangement reactions.

### Phase III: ring rearrangements

While C23 is still static in phase III, C20 is relatively flexible, which might serve to decrease the affinity for the enzyme’s binding pocket. Interestingly, although different types of ring rearrangement reactions occur in each pathway, the same inherent mobility trends were observed. As shown in Figure 3, C4–C11, C21 and C22 appear to be quite mobile in the terpene cyclase binding pocket in both pathways, indicating that their steric interaction with the enzyme has little influence on this phase of the biosynthesis. Based on this analysis, it appears that the destination of the cyclization cascade is determined by the conformations of the carbocation intermediates, but not by enzymatic constraints. This insight is consistent with our previous findings [1].

## Conclusion

In order to clarify the influence of the conformational preorganization [20] of GFPP bound to a sesterterpene synthase on the reaction outcome, we computed the inherent atomic mobility in the carbocation intermediates during the biosynthesis of two sesterterpenes, quiannulatene and sesterfisherol. Two methyl groups, i.e., C20 and C23, remain static during the first half of the cyclization cascade, indicating that they could be determinants of the affinity for the enzyme cavity of sesterterpene synthase. Interestingly, inherent mobility shows the same trend in phase I (5/12/5 tricycle formation) and phase III (ring rearrangement), but not in phase II (conformational changes and hydrogen shifts), of quiannulatene and sesterfisherol biosynthesis, indicating that phase II is the key process for the structural diversification, in accordance with our previous study [1]. Moreover, C20 becomes flexible in phase III, which could result in decreased affinity for the enzyme, and this might be relevant to substrate release. Few X-ray crystal structures of terpene cyclases are available [7–8], so we believe inherent mobility analysis will be useful to predict the mechanism of conformational preorganization of the substrate to achieve different biosynthetic outcomes in other terpene-forming reactions. We are currently applying this approach to sesterterpenes that are synthesized through a 5/6/11 tricyclic intermediate.

## Experimental

All calculations were carried out using GRRM11 [21–25] with Gaussian 09 [26]. All TS structures were obtained from the literature [1,3]. Intrinsic reaction coordinate (IRC) calculations [27–30] were carried out with B3LYP/6-31+G(d,p) for quiannulatene and with M06-2X/6-31G(d,p) for sesterfisherol. All 3D structures were drawn by Gauss View 6. All displacement calculations were performed using in-house software written in C++ (see Supporting Information File 1).

## Supporting Information

File 1 Coordinates and energies of quiannulatene biosynthesis.

File 2 Coordinates and energies of sesterfisherol biosynthesis.

File 3 Software for inherent mobility analysis.

File 4 How to use in house software to calculate inherent atomic mobility.
