# Supplementary material for: Inherent atomic mobility changes in carbocation intermediates during the sesterterpene cyclization cascade
Source: Beilstein J Org Chem. 2019 Aug 7;15:1890–7. doi: 10.3762/bjoc.15.184 (PMC6693403; doi:10.3762/bjoc.15.184)
Supplement: File 4 — How to use in house software to calculate inherent atomic mobility. [file Beilstein_J_Org_Chem-15-1890-s004.pdf]

BEILSTEIN JOURNAL OF ORGANIC CHEMISTRY

Supporting Information

for

Inherent atomic mobility changes in carbocation intermediates during the sesterterpene cyclization cascade

Hajime Sato, Takaaki Mitsuhashi, Mami Yamazaki, Ikuro Abe and Masanobu Uchiyama

Beilstein J. Org. Chem. **2019,** *15,* 1890–1897. doi:10.3762/bjoc.15.184

How to use in house software to calculate inherent atomic mobility

License and Terms: This is a supporting information file under the terms of the Creative Commons Attribution License (http://creativecommons.org/licenses/by/4.0). Please note that the reuse, redistribution and reproduction in particular requires that the authors and source are credited.
The license is subject to the *Beilstein Journal of Organic Chemistry* terms and conditions: (https://www.beilstein-journals.org/bjoc)

Table of Contents

1. How to use in house software to calculate inherent atomic mobility..S2

How to use in house software to calculate inherent atomic mobility

1. **The preparation of input file.**
   In this study, all IRC calculations were carried out using GRRM11. The GRRM's log files need to be converted to appropriate format as shown in S1.log and S2.log.

2. **Calculation of inherent atomic mobility.**
   Please compile S3.cpp as following.

   g++ S3.cpp –o S3

   Then, execute S3 as following.
   ./S3 [inputfile]
   This software generates two csv files; *output_all.csv* and *output_carbon.csv*.

3. **Analysis**
   Open the *output_carbon.csv* and make heat map using Microsoft Excel.
